# Targeted design and identification of AC1NOD4Q to block activity of HOTAIR by abrogating the scaffold interaction with EZH2

**DOI:** 10.1186/s13148-019-0624-2

**Published:** 2019-02-14

**Authors:** Yu Ren, Yun-fei Wang, Jing Zhang, Qi-xue Wang, Lei Han, Mei Mei, Chun-sheng Kang

**Affiliations:** 1 Department of Neurosurgery, Tianjin Medical University General Hospital, Lab of Neuro-oncology, Tianjin Neurological Institute, Key Laboratory of Post-Neuro injury Neuro-repair and Regeneration in Central Nervous System, Ministry of Education and Tianjin City, Tianjin, 300052 China; 2 Department of Genetics, School of Basic Medical Sciences, Tianjin Medical University, Tianjin, 300070 China; 3 Irving Cancer Research Center, Columba University, New York, 10032 USA; 4 Department of Cell Biology, School of Basic Medical Sciences, Tianjin Medical University, Tianjin, 300070 China; 5 Affiliated Cancer Hospital and Institute of Guangzhou Medical University, Guangzhou, 510095 China

**Keywords:** AC1NOD4Q, HOTAIR, EZH2, NLK, High-throughput screening

## Abstract

**Background:**

Nearly 25% of long intergenic non-coding RNAs (lincRNAs) recruit chromatin-modifying proteins (e.g., EZH2) to silence target genes. HOX antisense intergenic RNA (HOTAIR) is deregulated in diverse cancers and could be an independent and powerful predictor of eventual metastasis and death. Yet, it is challenging to develop small molecule drugs to block activity of HOTAIR with high specificity in a short time.

**Results:**

Our previous study proved that the 5′ domain, but not its 3′ domain, was the function domain of HOTAIR responsible for tumorigenesis and metastasis in glioblastoma and breast cancer, by recruiting and binding EZH2. Here, we targeted to establish a structure-based methodology to identify lead compounds of HOTAIR, by abrogating scaffold interactions with EZH2. And a small compound AC1NOD4Q (ADQ) was identified by high-throughput molecular docking-based virtual screening of the PubChem library. Our analysis revealed that ADQ was sufficiently and specifically interfering HOTAIR/EZH2 interaction, thereby impairing the H3K27-mediated tri-methylation of NLK, the target of HOTAIR gene, and consequently inhibiting tumor metastasis through Wnt/β-catenin pathway in vitro and in orthotopic breast cancer models. The results of RIP and EMSA further revealed that 36G46A of 5′ domain was the essential binding site for ADQ exerted its inhibitory effect, further narrowed the structure and function of HOTAIR from the 5′ functional domain to the micro-domain.

**Conclusions:**

Our findings suggest of a potential new strategy to discover the lead compound for targeted lincRNA therapy and potentially pave the way for exploiting ADQ as a scaffold for more effective small molecule drugs.

**Electronic supplementary material:**

The online version of this article (10.1186/s13148-019-0624-2) contains supplementary material, which is available to authorized users.

## Background

Epigenetic regulation has drawn remarkable attention, particularly regarding long non-coding RNAs (lncRNAs), which can fold into higher order structures to provide substantially greater potential for target recognition [1–3]. Nearly 25% of lincRNAs associate with polycomb repressive complex 2 (PRC2) to facilitate chromatin remodeling as well as transcriptional and post-transcriptional regulation [4–6]. And one of the deepest and broadest studied PRC2 interacting lncRNA is HOTAIR, which is deregulated in diverse cancers and could be an independent and powerful predictor of eventual metastasis and death [7, 8]. However, developing a structure-based methodology to identify lead compounds that specifically blocking the activity of HOTAIR is a great demand but still challenging.

HOTAIR serves as a modular scaffold of histone modification complexes, with its 5′ domain binding the PRC2 complex and 3′ domain binding the LSD1/CoREST complex to silence specific gene expression [9]. Recent studies indicated that HOTAIR promoted the invasion of breast carcinoma cells in a PRC2-dependent manner [10, 11]. Our previous study proved that the 5′ domain, but not its 3′ domain, which was the function domain of HOTAIR is responsible for tumorigenesis and metastasis in glioblastoma and breast cancer, by recruiting and binding EZH2, the catalytic subunit of PRC2, to silence target genes nemo-like kinase (NLK) [12–14]. And 212–300 nucleotides (nt) of HOTAIR were further determined as the minimal and pivotal EZH2-binding element [15].

**Fig. 1:**
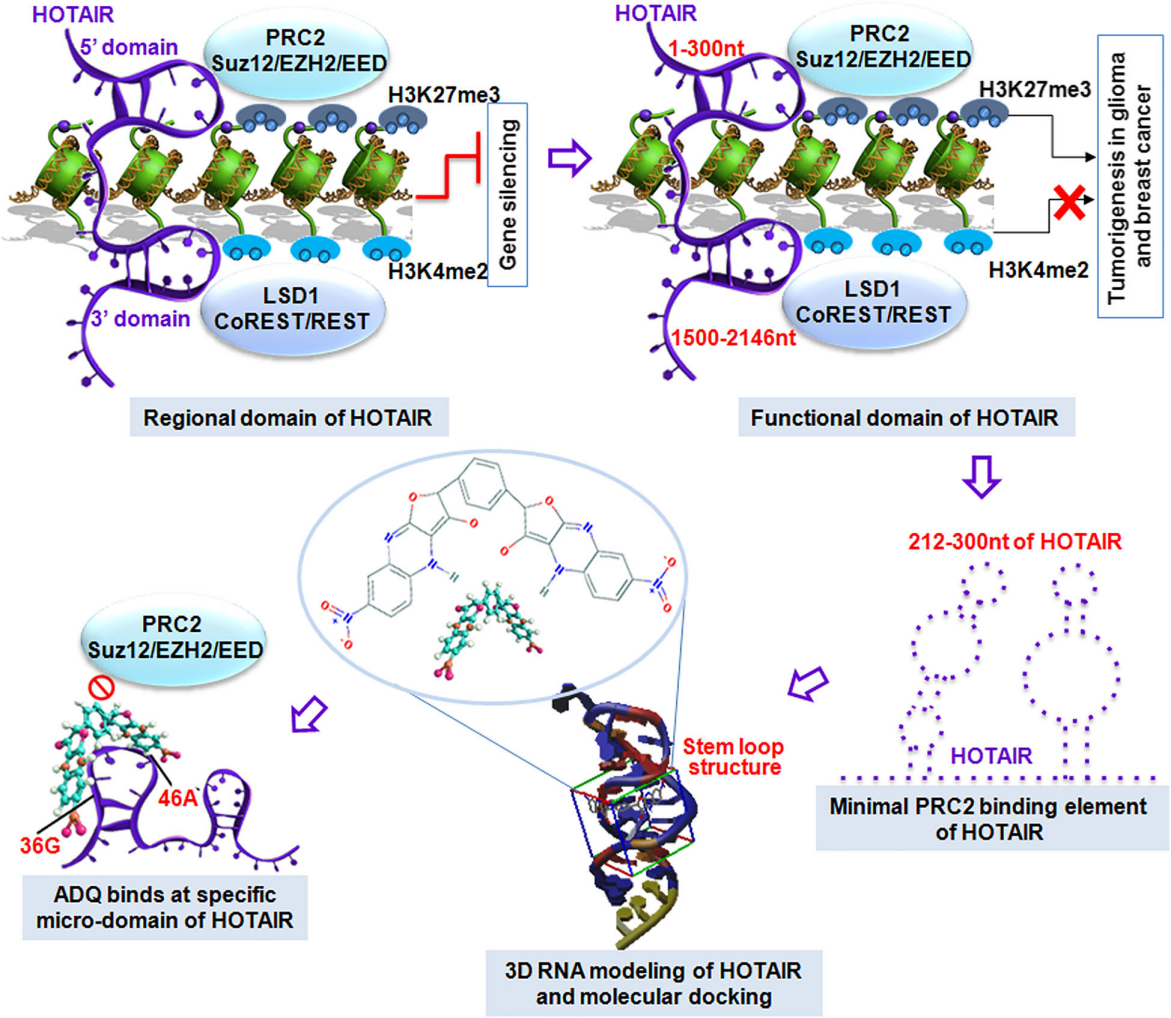
Schematic illustration of studying the structure and function of HOTAIR and identifying AC1NOD4Q as a small molecule inhibiting the HOTAIR/PRC2 complex interaction. The 5′ domain, but not the 3′ domain, proved to be the functional domain responsible for tumorigenesis in glioma and breast cancer. Further analysis demonstrated that the minimal HOTAIR domain required for PRC2 binding is 212–300 nt. Using 3D modeling prediction, we found that this domain contains several hairpin loop structures and serves as a target for small molecule intervention. By performing in silico high-throughput screening, we determined that AC1NOD4Q binds at a specific HOTAIR micro-domain (36G46A) and induces strong molecular inference in the scaffold interaction between the PRC2 complex and HOTAIR

Based on deep understanding of the structure and function of HOTAIR, here, we aimed to establish a structure-based method by performing in silico high-throughput screening to identify a lead compound for molecular inference in the HOTAIR/EZH2 scaffold interaction and targeted HOTAIR therapy (Fig. 1). Using 3D modeling prediction by MC-Fold and MC-Sym programs, we found that this domain contains several hairpin loop structures and serves as a target for small molecule intervention. Indeed, our previous work has successfully discovered a specific small-molecule inhibitor of miR-21, named AC1MMYR2, by modeling the 3D structure of the pre-miR-21 Dicer-binding site using the same program [16]. Numerous compounds were identified via screening the PubChem library, and AC1NOD4Q (ADQ) was selected as the lead compound due to its desirable potency and physicochemical properties. By binding to the specific 36G46A micro-domain of HOTAIR, ADQ efficiently abrogate the HOTAIR/EZH2 interaction, thereby inhibiting H3K27-mediated tri-methylation of the NLK and consequently inhibiting tumor metastasis. Our findings established a novel methodology to develop new classes of small molecule drugs by screening libraries of synthetic compounds and natural products, with different binding sites and higher binding affinities, potentially paving the way for exploiting ADQ as a scaffold for more effective small molecule drugs.

## Results

### Identification of AC1NOD4Q (ADQ) as the potent compound

**Fig. 2:**
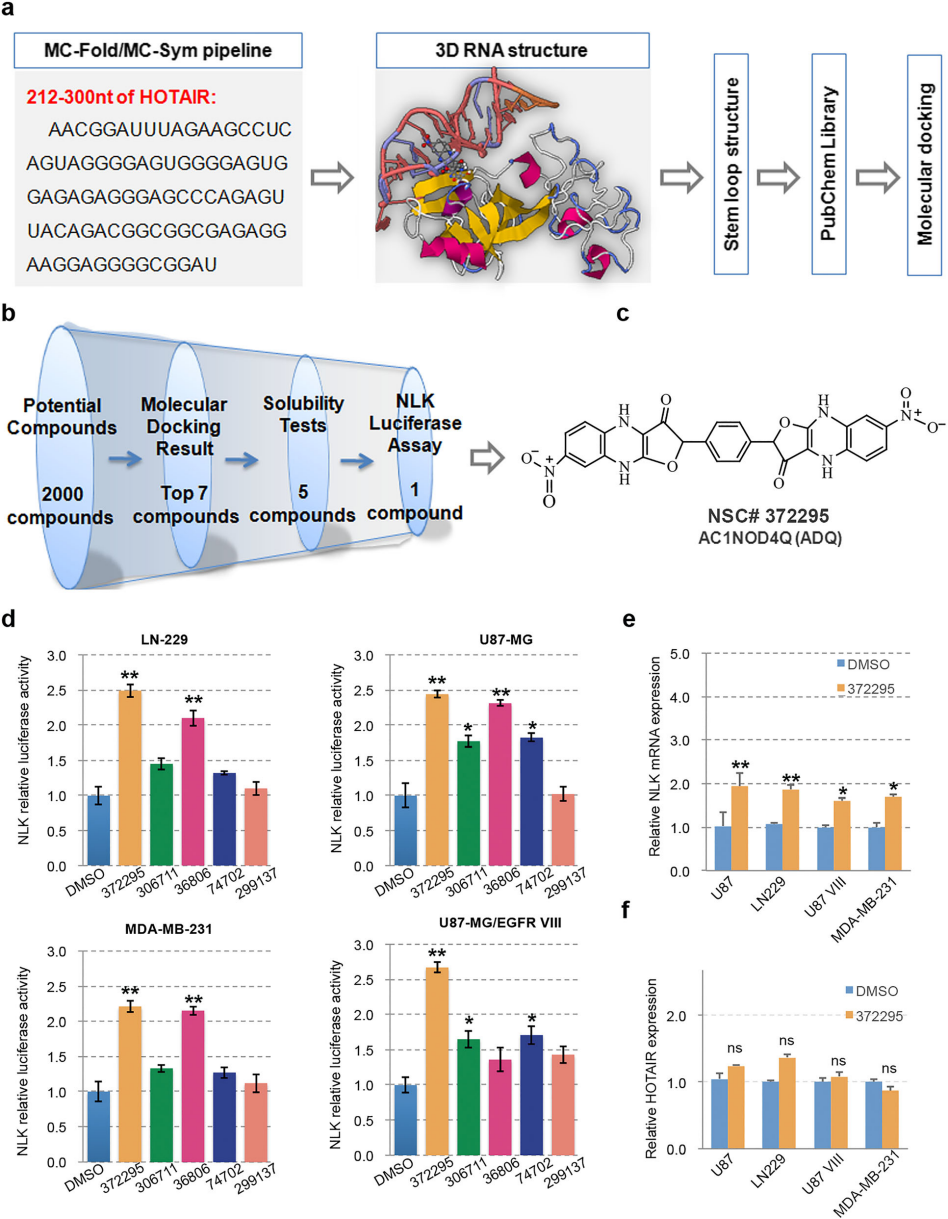
3D HOTAIR modeling prediction and identification of AC1NOD4Q as a potent compound interfering the HOTAIR/EZH2 interaction. **a** 3D RNA modeling of 212–300 nt in the 5′ functional domain of HOTAIR that binds to EZH2 was conducted by the MC-Fold and MC-Sym programs. **b** Screening process to identify lead compounds that specifically abrogate scaffold interactions between the PRC2 complex and HOTAIR. **c** Chemical structure of ADQ. **d** ADQ treatment had the strongest effect on NLK luciferase activation in LN229, U87, and U87 EGFRvIII glioma cells and MDA-MB-231 breast cancer cells. **e** NLK mRNA levels were measured in various cell lines via qRT-PCR after treatment with ADQ. **f** HOTAIR expression was detected in DMSO- and ADQ-treated cells

Based on deep understanding of the structure and function of HOTAIR/EZH2 interaction, the sequence data (212–300 nt) were used to construct 3D models using the MC-Fold and MC-Sym programs, allowing prediction of the 3D hairpin loop structure within the PRC2-binding element of HOTAIR [17]. The tertiary structure of the minimal PRC2-binding domain of HOTAIR contains several hairpin loop structures, which define protein RNA-binding sites, providing an ideal model for compound interference. High-throughput molecular docking-based virtual screening of the PubChem library was conducted using the AutoDock program (Fig. 2a). Details of the structure-based design effort were reported in our previous study [16]. The binding energy was calculated by AutoDock program and optimized using TINKER software (Additional file 1: Table S2).

From the initial screen, we identified 7 potential compounds with high binding affinity that possessed free energies greater than 9.8 (Fig. 2b). However, two of the potential compounds were not soluble in water, DMSO or ethanol. In the previous study, NLK was proven to be a transcriptional target of HOTAIR [14]. Thus, the NLK luciferase activity of the remaining five compounds was then determined.

Among the five compounds, ADQ (7-nitro-2-[4-(7-nitro-3-oxo-4, 9-dihydrofuro[3, 2-b] quinoxalin-2-yl)phenyl]-4, 9-dihydrofuro[3, 2-b]quinoxalin-3-one) exhibited the strongest binding affinity for HOTAIR (binding energy, 10.5). The chemical structure of ADQ is shown in Fig. 2c and Additional file 1: Figure S1. Compared to the other compounds, ADQ displayed the highest NLK luciferase activity in four human epithelial cancer cell lines (U87, U87 EGFRvIII, and LN229 glioblastoma cells and MDA-MB-231 breast cancer cells, Fig. 2d). Additionally, NLK mRNA levels were increased by 1.5–2.0-fold in ADQ-treated cells (Fig. 2e). ADQ did not alter the expression of HOTAIR (Fig. 2f), indicating that this molecule does not directly regulate HOTAIR expression. Based on these results, ADQ was selected as the lead compound among the candidates.

### ADQ selectively interrupts the HOTAIR/EZH2 interaction

**Fig. 3:**
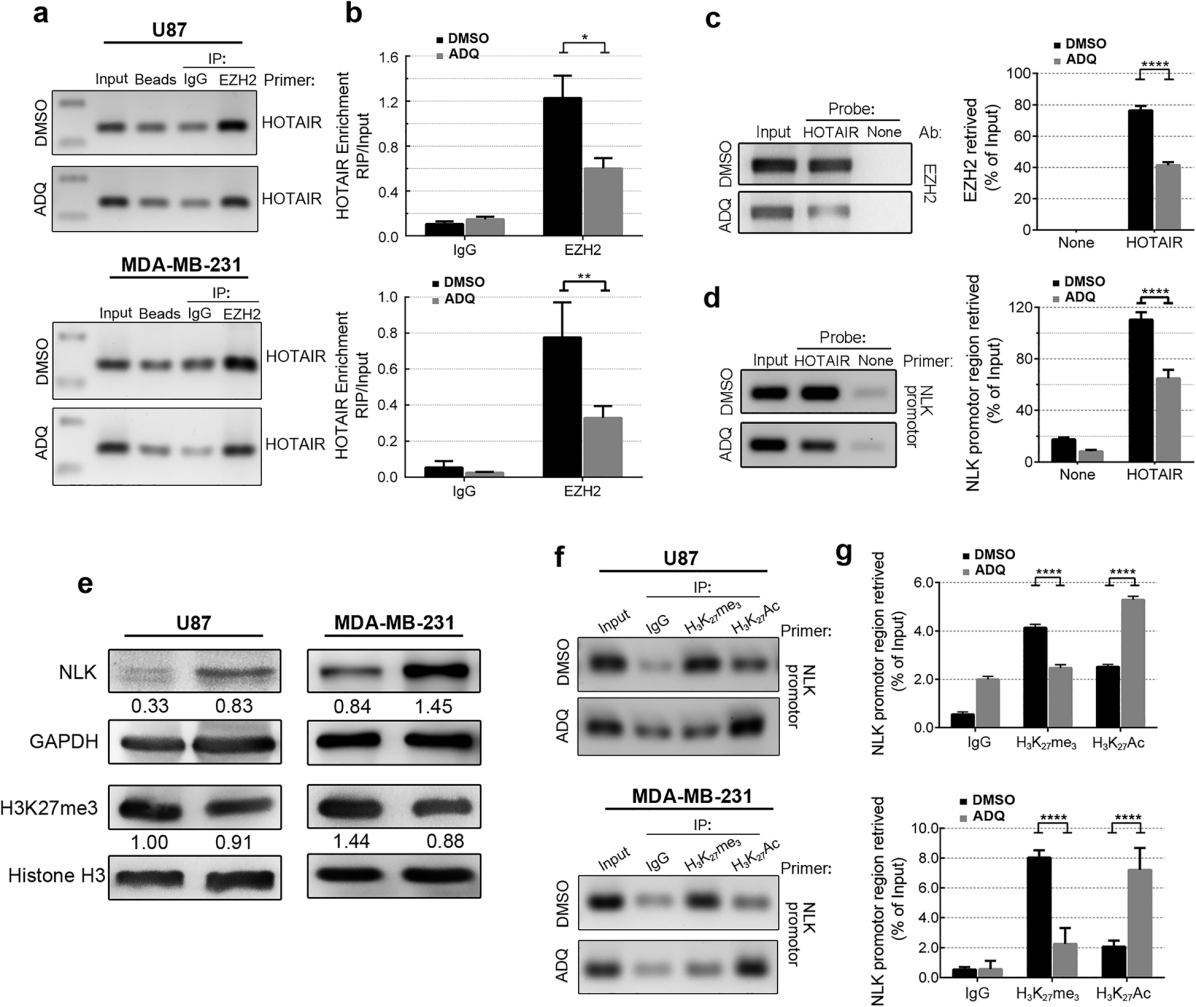
ADQ blocks the HOTAIR/EZH2 interaction and inhibits the H3K27-mediated tri-methylation of NLK. **a** RIP assays were performed to detect the binding efficiency of the HOTAIR fragment in combination with EZH2 proteins. **b** RNA levels in immunoprecipitants were determined by qRT-PCR and expressed as the relative fold enrichment after subtracting the matched IgG negative control. **c**, **d** ChIRP was used to assess the binding activity of HOTAIR with EZH2 and the NLK promoter in control and ADQ-treated U87 and MDA-MB-231 cells. **e** NLK and H3K27me3 protein levels were detected by Western blotting. **f** ChIP was performed with anti-H3K27me3 and anti-H3K27Ac antibodies in cells treated with DMSO or ADQ, and anti-IgG served as the negative control. Chromatin obtained by pull-down was used for qPCR to amplify the promoter region of NLK located 2 kb upstream of the transcription start site. **g** Fold enrichment of H3K27me3 and H3K27Ac on the NLK promoter normalized to the IgG signal after normalization to the respective input signal

To test the performing function of this compound, RNA immunoprecipitation (RIP) analysis using EZH2 antibody was first performed. By isolating the specific protein-bound HOTAIR, RNA sample was extracted and determined by agarose gel electrophoresis and qPCR. As shown in Fig. 3a, both U87 and MDA-MB-231 cells treated with ADQ exhibited significantly reduced HOTAIR-binding EZH2 signals. And the PCR results demonstrated that the HOTAIR levels bound to EZH2 were reduced to 2-fold after ADQ treatment, indicating that ADQ prevented the formation of the HOTAIR/EZH2 complex (Fig. 3b). Indeed, the binding efficiency of 5 other lncRNAs in combination with EZH2 proteins, including MALAT1, HOTAIRM1, KCNQ1OT1, HOXA11, and XIST fragment, which were reported to be able to bind PRC2 complex, was also detected. ADQ treatment did not show any significant reduction in none of the five lincRNA signals compared to the control group (Additional file 1: Figure S2).

ChIRP-qPCR was further employed to assess the binding activity of HOTAIR with EZH2 and NLK. After in vivo cross-linking and chromatin fragmentation, the biotinylated probe was hybridized complementary to HOTAIR, and bound chromatin was isolated*.* EZH2 binding was dramatically reduced after ADQ treatment (Fig. 3c). Moreover, analysis of co-purified genomic DNA by qPCR detected a reduced occupancy on the NLK promote after ADQ treatment, indicating that ADQ affected the binding of HOTAIR to the target gene (Fig. 3d).

Epigenetic processes, including promoter DNA methylation and transcript silencing, by lncRNAs were recently shown to be involved in tumorigenesis and cancer progression [18, 19]. Our previous study demonstrated that HOTAIR-mediated H3K27 tri-methylation was responsible for decreased NLK expression, which contributed to activation of the β-catenin signaling pathway [14]. Western blot analysis revealed that reduced H3K27me3 expression and elevated NLK protein expression levels were detected in both ADQ-treated cell lines (Fig. 3e). Moreover, ChIP analysis detected a marked reduction in H3K27-mediated tri-methylation in the NLK promoter region in ADQ-treated cells (Fig. 3f, g).

Collectively, these results suggested that AC1NOD4Q was a potent and selectively compound interfering the EZH2/HOTAIR interaction identified by 3D HOTAIR structure-based methodology modeling and high-throughput screening.

### ADQ specifically binds HOTAIR at 36G46A micro-domain

**Fig. 4:**
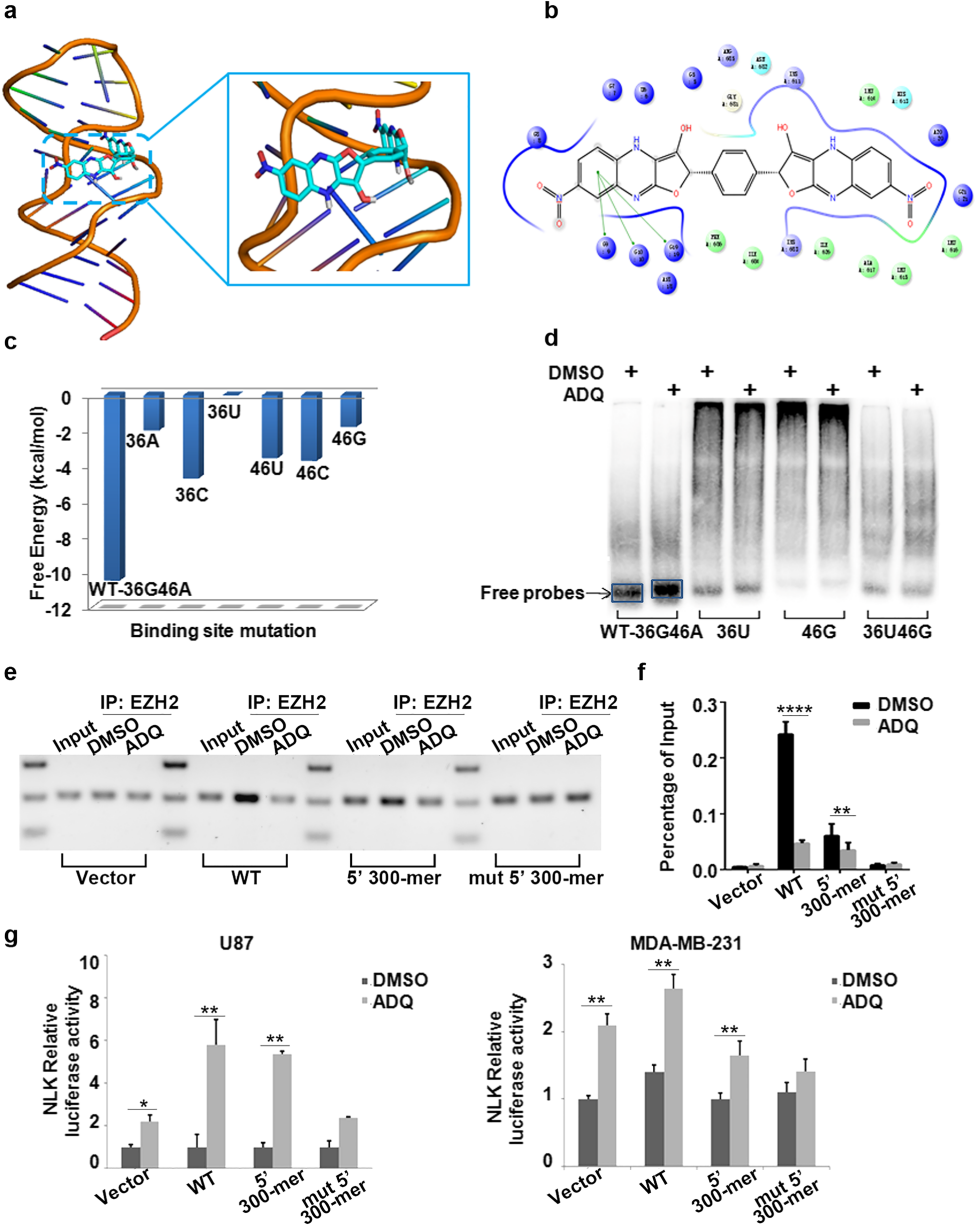
ADQ binds to the 36G46A sequence of HOTAIR. **a**, **b** Molecular docking model of ADQ bound to HOTAIR to predict the specific binding site. **c** Binding energy analysis revealed that 36G46A resulted in the strongest free energy and binding stability. A mutation in either sequence greatly reduced the binding energy. **d** EMSA was performed using 5′ biotin-labeled HOTAIR probes to detect the HOTAIR/ADQ binding activity in the wild-type and mutant groups. **e** HOTAIR full-length (WT), 5′ domain (5′ 300-mer), and 5′ mutation domain (mut 5′ 300-mer) constructs were stably transfected into U87 cells. RIP assays detected the binding efficiency of the HOTAIR fragment in combination with EZH2 proteins in various group. **f** RNA levels in immunoprecipitants were determined by qRT-PCR and expressed as the relative fold enrichment after subtracting the matched IgG negative control. **g** ADQ increased the NLK luciferase activity in U87 WT and 5′ domain cells, whereas ADQ had little effect on 5′ domain with 36G46A mutation cells. A similar pattern was observed in MDA-MB-231 cells

To study the specific binding site of ADQ, we evaluated the binding affinity in 89 (212–300 nt) HOTAIR base pairs by conformation analysis and molecular docking model using the AutoDock program. The distance and their interaction were the most critical factors influencing the binding affinity between ADQ and HOTAIR base pair. Basing on the above analysis, 36G46A resulted in the lowest free energy and were identified as the specific binding site. The interactions formed between ADQ and HOTAIR are illustrated in Fig. 4a. Specifically, one nitrobenzene fragment of ADQ formed *π*-*π* stacking with the 36G sequence, while another nitrobenzene fragment inserted the binding pocket near the 46A sequence (Fig. 4b). Furthermore, some mutations in silico were performed to verify the binding site and we found that any mutation in 36G or 46A could significantly increase the calculated binding energy, thereby reducing the binding stability between HOTAIR and ADQ (Fig. 4c).

To verify the micro-domain of ADQ binding to HOTAIR, EMSA was performed with 5′ biotin-labeled HOTAIR 36G46A wild-type probes and 36U46A, 36G46G, and 36U46G mutant probes. Because of the low molecular weight of the compound, adding ADQ to the wild-type HOTAIR probe induced a moderate gel shift, indicating that ADQ directly bound HOTAIR (Fig. 4d). ADQ treatment increased signals of disassociation wild type HOTAIR probe with EZH2 protein. However, ADQ treatment markedly attenuated the increased percentage of the mutant HOTAIR probe, further confirming the computational results.

To further confirm the functional domain of ADQ, HOTAIR full-length (WT), 5′ domain (5′ 300-mer), and 5′ domain with 36U46G mutation (mut 5′ 300-mer) constructs were stably transfected into U87 and MDA-MB-231 cells. Upon HOTAIR immunoprecipitation and SDS-PAGE, immunoblotting with an EZH2 antibody revealed that EZH2 interacted with both the wild-type 36G46A and mutant 36U46G HOTAIR sequences. ADQ diminished the binding efficiency of the HOTAIR fragment in combination with EZH2 proteins in WT and 5′ domain transfected cell, but did not affect the mutant type (Fig. 4e, f). Moreover, the NLK luciferase activity was significantly enhanced in ADQ-treated WT and 5′ domain cells, whereas ADQ exerted a minimal effect on 5′ domain with 36U46G mutation cells (Fig. 4g). Taken together, 36G46A of 5′ domain was the essential binding site for ADQ exerting its inhibitory effect.

### Structure-activity relationship

**Fig. 5:**
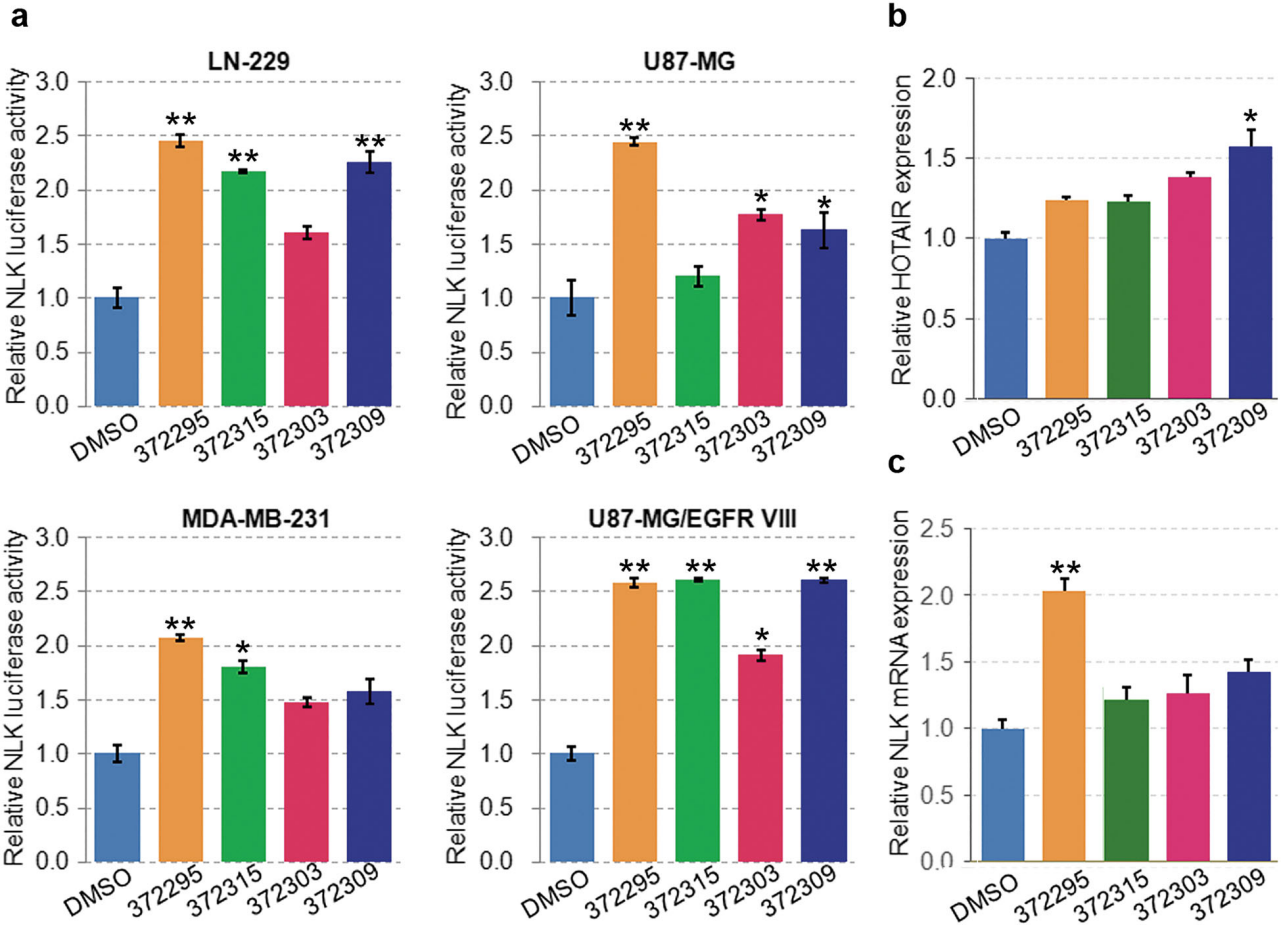
Structure-activity relationship analysis to elucidate the critical ADQ residues responsible for the inhibitory binding activity of HOTAIR and EZH2. **a** NLK luciferase activity was measured in LN229, U87, and U87 EGFRvIII glioma cells and in MDA-MB-231 breast cancer cells. **b**, **c** HOTAIR expression and NLK mRNA levels were detected after treatment with several structural analogs of ADQ in U87 cells

To explore the structural elements of ADQ that are critically involved in abrogating the interaction between HOTAIR and EZH2, we conducted a structure-activity relationship analysis using several structural analog compounds (Additional file 1: Table S2). As shown in Fig. 5a, compound NSC#372315 increased the NLK luciferase activity to some extent, while none of the three compounds significantly increase NLK mRNA levels (Fig. 5b, c). Thus, the structure-activity relationship analysis of 2, 2′-benzene-1, 4-diylbis(4, 9-dihydrofuro[2, 3-b]quinoxalin-3(2H)-one) provided information on the critical residues responsible for the inhibitory interactions between the PRC2 and HOTAIR. The nitro substitution was important for maintaining its inhibitory activity, whereas chloro and dimethyl substitutions resulted in a loss of inhibition, suggesting that the nitro group has a significant effect. According to our predicted binding model (Fig. 4a), *π*-*π* stacking formed between ADQ and HOTAIR. Among these four analogs, the nitro group enhanced *π*-*π* stacking, as the nitrobenzene fragment had a more substantial conjugated effect than the other substituted groups in NSC372303, NSC372309, and NSC372315. Based on these results, we speculated that *π*-*π* stacking plays a key role in the ADQ functions on HOTAIR.

### ADQ inhibits cell invasion and migration by blocking the β-catenin signaling pathway

**Fig. 6:**
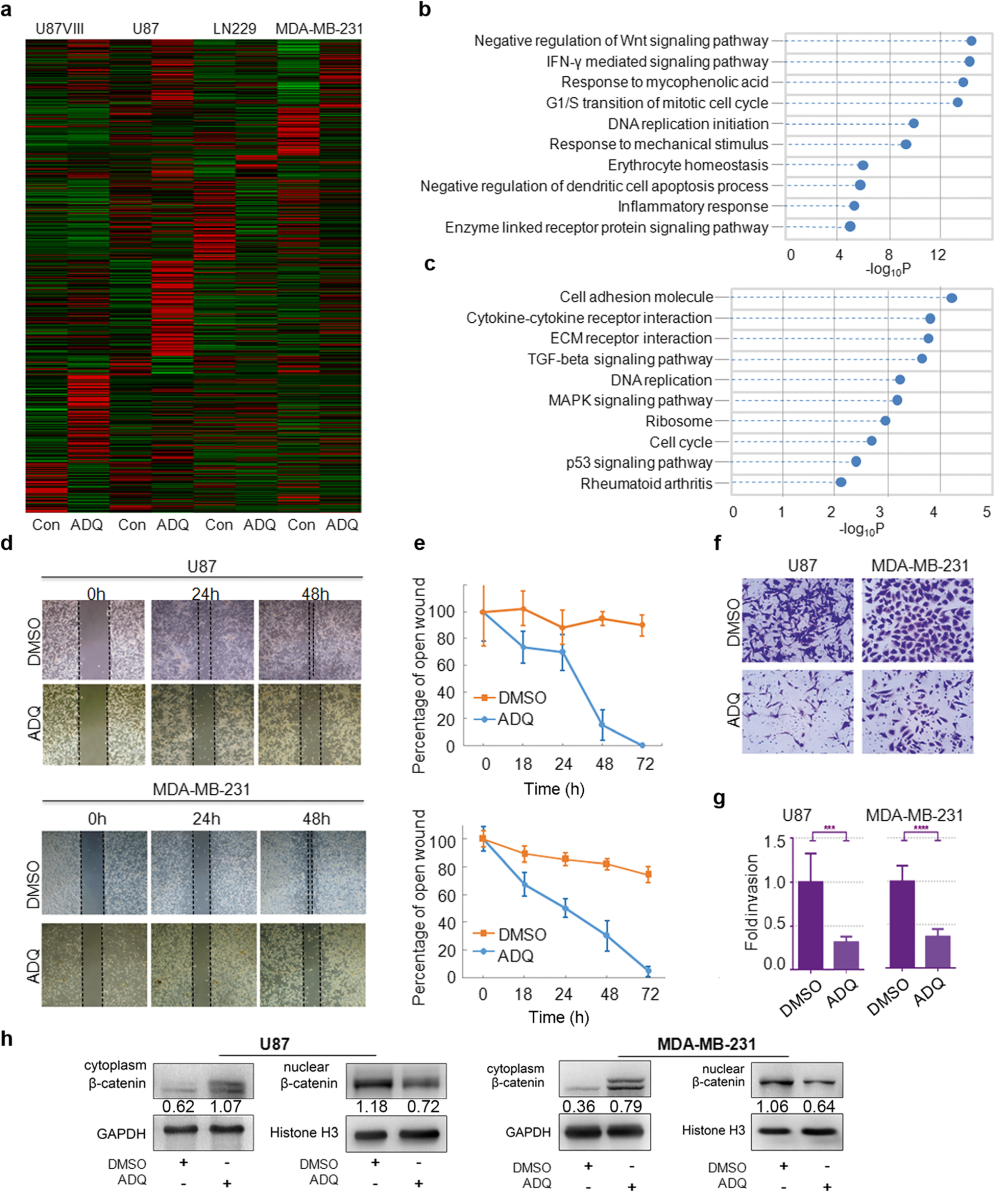
Computational analysis of significantly dysregulated mRNAs revealed that ADQ is involved in tumor migration and metastasis. **a** MRNA microarrays were performed to identify differentially expressed genes in cells treated with DMSO or ADQ. **b**, **c** GO biology process and KEGG pathway enrichment analyses were performed, which indicated that the anti-cancer effects of ADQ may involve the repression of cell migration and metastasis. **d**, **e** The wound healing assay revealed that cell migratory activities were significantly reduced after treatment with ADQ. **f**, g ADQ treatment decreased the number of invaded cells, as indicated by the transwell assay. **h** ADQ treatment inhibited the β-catenin signaling pathway. The protein levels of nuclear β-catenin were significantly decreased in ADQ-treated cells

To clarify the biological function of ADQ, mRNA microarrays were performed in three randomly selected samples from each group (Fig. 6a). Among the four cell lines, MDA-MB-231 cells exhibited the clearest and remarkable changing trend, and we thus focused on this cell line in subsequent analysis. Extensive differentially expressed genes (DEGs) were identified. 90 upregulated and 64 downregulated mRNAs (fold change ≥ 2.0, *P* < 0.05) existed in ADQ-treated MDA-MB-231 cells compared with the control group.

To further explore the distribution of DEGs, GO enrichment analysis was performed. As shown in Fig. 6b, a wide range of biological processes was affected. Importantly, “Negative regulation of Wnt signaling pathway” was the top-ranked biological process. The top 10 KEGG pathways in the dysregulated mRNAs are shown in Fig. 6c, including “cell adhesion molecule,” “cytokine-cytokine receptor interaction,” “ECM receptor interaction,” and “TGF-β signaling pathway.” Thus, we hypothesized that ADQ contributes to the inhibition of aggressive tumor behavior by inactivating cell adhesion molecules and the epithelial-to-mesenchymal transition (EMT), suggesting a tight link between ADQ and biological processes involving tumor migration and metastasis.

In the wound healing assays, the migratory activity of ADQ-treated cells was clearly reduced (Fig. 6d, e). The transwell assay results suggested that ADQ treatment decreased the number of invaded cells by 3- to 5-fold compared with that in control cells (Fig. 6f, g).

Aberrant activation of β-catenin is a critical driver in the development and progression of EMT [20, 21]. Our previous study demonstrated that knocking down HOTAIR efficiently repressed the activity of the β-catenin signaling pathway. Thus, we next determined whether ADQ affected β-catenin activity. Western blot analysis indicated that β-catenin expression in the whole-cell lysate, nucleus, and cytosolic lysate was remarkably decreased in ADQ-treated cells (Fig. 6h). Therefore, we hypothesized that ADQ contributes to the inhibition of aggressive tumor behavior via the inactivation of cell adhesion molecules and EMT.

### ADQ inhibits the growth and metastasis of xenograft tumors

**Fig. 7:**
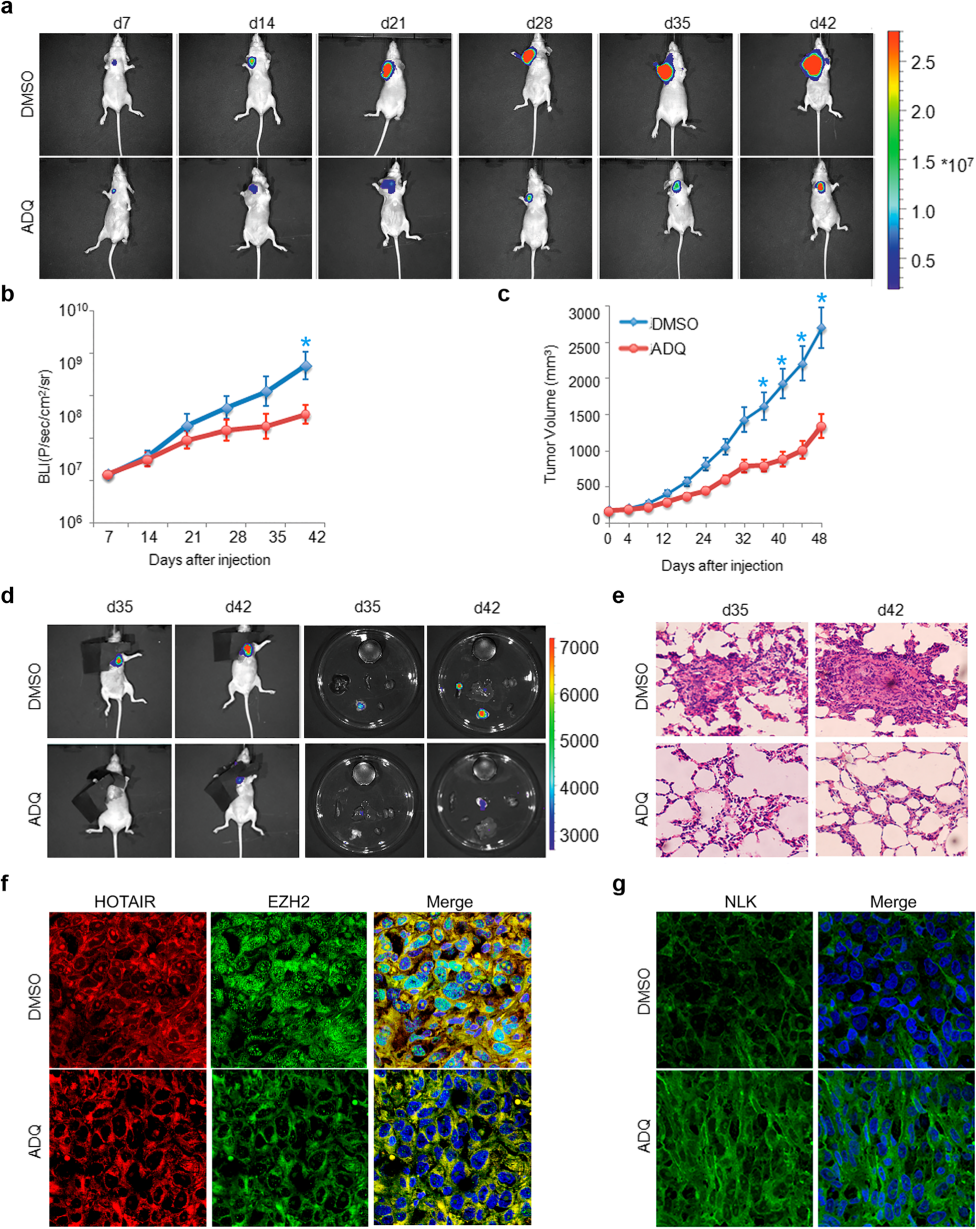
ADQ inhibits the growth and metastasis of xenograft tumors. **a** Representative pseudocolor bioluminescence images of mice on days 7, 14, 21, 28, 35, and 42. Mice bearing orthotopic MDA-MB-231 tumors were intraperitoneally injected with PBS or 15 mg/kg ADQ every 2 days. Each group included eight mice (*n* = 8). **b** Representative bioluminescence images (BLIs) of PBS- and ADQ-treated tumors. **c** Tumor volume curves were evaluated. **d** BLIs of lung metastasis on days 35 and 42. **e** Representative photomicrographs of H&E-stained lung tissues. **f** Fluorescence in situ hybridization using 5′ DIG-labeled HOTAIR probes and FITC-labeled EZH2 antibody was performed to detect the HOTAIR/EZH2 binding activity. **g** NLK expressions were examined by immunohistochemistry in tumor sections

Motivated by the specific and inhibitory efficacy of ADQ in vitro, we further explored its anti-tumor effects in vivo using an MDA-MB-231 orthotopic tumor transplantation model in nude mice. A significant reduction in tumor growth was detected in the ADQ-treated mice (Fig. 7a, c). More importantly, ADQ treatment resulted in similar cell growth and lung metastasis inhibition as that in the HOTAIR knockdown group (Additional file 1: Figure S3). Furthermore, lung metastasis in ADQ-treated mice was dramatically lower than that in control mice (Fig. 7d). Injection of mice with MDA-MB-231 cells resulted in the formation of large metastatic nodules in the lungs. In contrast, the number and volume of metastatic nodules in the lung of mice injected with ADQ were significantly reduced as compared with that injected with MDA-MB-231-control cells (Fig. 7e), indicating that ADQ efficiently represses HOTAIR-mediated tumor metastasis.

In addition, we comparatively studied the HOTAIR/PRC2 interaction through RNA in situ hybridization and immunofluorescence so as to unveil the function of ADQ in vivo. As shown in Fig. 7f, confocal microscopy revealed co-localization signals between Cy3 and FITC fluorescence in the nucleus and cytoplasm, suggesting that HOTAIR has a strong binding affinity with EZH2 in the control group. While ADQ treatment induced reduced and redistribution of EZH2 signals from nucleus to cytoplasm and disassociation of HOTAIR, indicating that ADQ could efficiently interfere the HOTAIR/PRC2 interaction in vivo. Moreover, immunohistochemistry staining showed the increased NLK-positive cells in the ADQ, demonstrating that ADQ could effectively blocked the HOTAIR/EZH2 interaction and recruitment of EZH2 to target genes, thereby elevating the expression of NLK, which further confirmed us in vitro study (Fig. 7g).

**Fig. 8:**
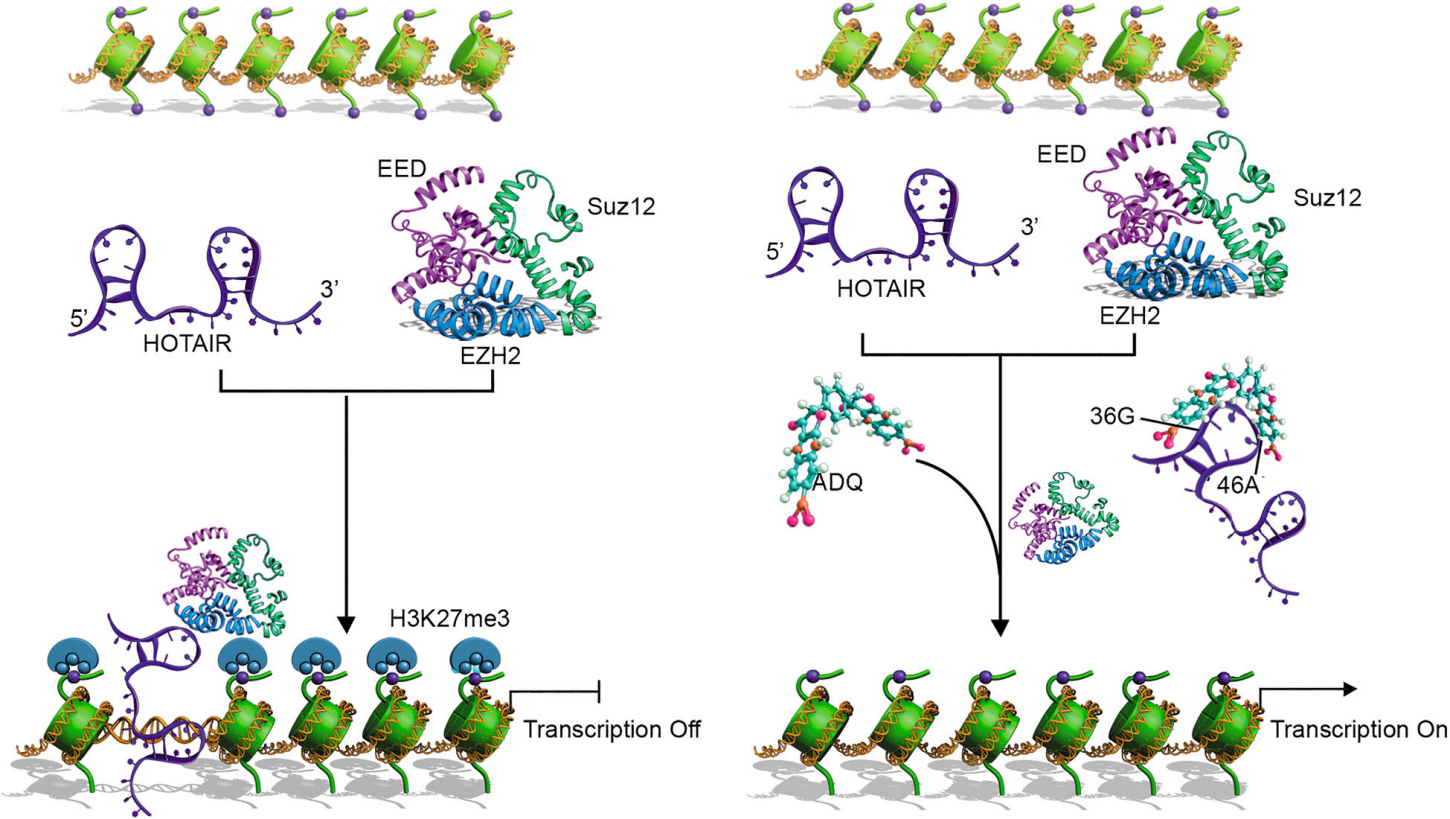
Schematic representation of the working mechanism of AC1NOD4Q. By binding to the 36G46A sequence in the HOTAIR5′ domain, ADQ weakens the recruitment and binding abilities of EZH2 and strongly inhibits the H3K27-mediated tri-methylation of the NLK promoter, thereby restoring its expression

Taken together, due to its high affinity for HOTAIR, ADQ treatment efficiently blocked the HOTAIR/EZH2 interaction via the 36G46A binding site, thereby reversing HOTAIR-induced cancer progression and metastasis (Fig. 8).

## Discussion

It is now evident that whereas less than 2% of the genome encodes proteins, at least 75% is actively transcribed into non-coding RNAs [22]. Therefore, considerable effort has been given to the development of the lead compounds for modulating the levels of non-coding RNAs for anti-cancer purposes. Specific knockdown using RNAi-based gene therapy has been explored, but the effective delivery of such agents into target tissues remains a major hurdle for nucleic acid-based therapies [23, 24].

Traditional drug discovery and development is based on measuring the activity of a large number of compounds, which is an interdisciplinary, expensive, and time-consuming process. With deep understanding of the structure and functional mechanisms of RNA, computer-aided drug design holds great promise in cancer therapy. The greatest advantage of structure-based drug design method lies in its “more wood behind fewer arrows.” That is to say, based on the structure and interaction of the target genes, this method could high-throughput screen the potential compounds in the shortest time by computer-aided drug design, thereby speeding up new target selection through the identification of hits to the optimization of lead compounds in the drug discovery process. Roos et al. discovered *N*-methyl-*N*-[3-(3-methyl [1, 2, 4] triazolo [4, 3-b]pyridazin-6-yl)phenyl] acetamide, which blocked the interaction between the RNA binding protein Lin28 and let-7, thereby rescuing let-7 processing and function [25]. Arenz et al. demonstrated that kanamycin bound to the pre-miR of let-7 and blocked Dicer activity [26, 27]. Our previous study identified that AC1MMYR2 could modulate miR-21 processing by specifically blocking the Dicer processing of pre-miR-21 into mature miR-21 [16].

Although lots of miRNA inhibitors have been identified, the study of compound screening for modulating lncRNA function is quite limited. The study of structure and function of the lincRNA enlightened us that this minimal binding domain of HOTAIR and EZH2 contains several hairpin loop structures, which serves as a target for small molecule intervention. And this may pave the way for the development of new drugs and targeted therapies to treat patients with glioma and breast cancer by blocking HOTAIR/PRC2 interaction.

And we identified that ADQ as a potent compound by performing in silico high-throughput screening. ADQ specifically bound the 36G46A site in the 5′ functional domain of HOTAIR (Fig. 4) and efficiently disrupted the interaction between HOTAIR and EZH2 both in vitro and in orthotopic tumor mouse models, thereby reducing the H3K27-mediated tri-methylation of NLK and consequently inhibiting tumor metastasis (Fig. 7). Indeed, other target gene of HOTAIR/EZH2, such as ZXH2 was also found in the gene expression array data, further demonstrating that ADQ could effectively blocked the HOTAIR/EZH2 interaction and recruitment of PRC2 to target genes (Additional file 1: Figure S3). Furthermore, ADQ did not alter the expression of HOTAIR, demonstrating that this molecule does not directly destabilize HOTAIR (Fig. 2f). Thus, our study clarifies the working mechanism of ADQ and further narrowed the structure and function of HOTAIR from the 5′ functional domain to the micro-domain, which is the novelty and the key point of the current paper.

Due to the dosage of ADQ used herein, this compound may not be sufficiently potent for clinical use, but it does provide a structural-based methodology that can be used to develop and identify lead compounds for molecular inference in scaffold interactions between the PRC2 complex and HOTAIR in cancer biology.

## Conclusion

LincRNA/EZH2-based targeting therapy may represent an alternative and complementary strategy in lincRNA/EZH2-dependent human cancers. Based on the structure and function of the HOTAIR, we targeted to establish a structure-based methodology by abrogating HOTAIR/EZH2 interactions and identify a lead compound of HOTAIR by binding at micro-domain 36G46A, thereby benefiting these glioma and breast cancer patients with high levels of HOTAIR and EZH2. Our findings provide a methodology to screen more larger libraries of synthetic compounds and natural products to identify other lead compounds for molecular inference in the scaffold interaction between the PRC2 complex and long non-coding RNAs in cancers.

## Methods

### Construction of the HOTAIR 3D structure

The full-length sequence of human HOTAIR was deposited in GenBank (bankit841140). Its 3D hairpin loop structure was predicted from sequence data using the MC-Fold/MC-Sym pipeline, an effective RNA structure determination approach, as previously described [17]. Energy optimization was further evaluated using the TINKER molecular modeling package (http://dasher.wustl.edu/tinker/).

### Molecular docking

The National Cancer Institute (NCI) diversity dataset (https://dtp.cancer.gov/organization/dscb/obtaining/default.htm/ diversity_explanation.html), which containedapproximatedly 200,000 compounds, was used as the small molecule library. Small molecule.pdb files were prepared using the prepare_ligand.py script. High-throughput docking-based virtual screening was conducted using the AutoDock program version 4. The rotational bonds of the compound were treated as flexible, whereas the RNA structure was kept rigid. Grid boxes were fixed around the Dicer binding site as the grid box center. RNA-ligand interactions were analyzed and visualized using Jmol (http://jmol.sourceforge.net/).

### Cell lines and culture conditions

Human epithelial cancer cell lines (U87 and LN229 glioma cells and MDA-MB-231 breast cancer cells) were obtained from American Type Culture Collection (ATCC). U87-MG cells stably transfected with EGFRvIII (U87 MG EGFRvIII, U87vIII) were provided by Prof. Huan Ren of the Department of Immunology at Harbin Medical University [28]. The cells were cultured at 37 °C in a 5% CO_2_ humidified incubator and maintained in Dulbecco’s modified Eagle’s medium (DMEM) supplemented with 10% fetal bovine serum (FBS) and 5% penicillin-streptomycin. Cells in the logarithmic growth phase or at 80% confluence were used for subsequent experiments.

### High-throughput screening

To screen the potential compounds for inhibiting the HOTAIR/EZH2 complex interaction, cells stably transfected with the NLK promoter firefly luciferase reporter were seeded onto 96-well plates and incubated at 37 °C overnight. Cells were treated with each small-molecule compound or DMSO for 48 h. Luciferase activity was measured using the FLx800 fluorescence reader and normalized to the negative controls. Compounds exhibiting a 1.5-fold increase compared with DMSO alone were considered active compounds. All small molecules were evaluated in triplicate.

### Gene network analysis

Three RNA samples from each group were randomly selected and sent to the Beijing Genomics Institute for microarray analysis. For mRNA analysis, GO, which describes genes from any organism and covers the domains of biological process (BP), cellular component (CC), and molecular function (MF), was used. Pathway analysis was performed to map genes to KEGG pathways. Fisher’s exact/chi-squared tests and FDR were used to determine significance, denoted by the *p* value between the GO term and the pathway correlated to the conditions. Smaller false discovery rates (FDRs) indicated smaller errors in judging the *p* value.

### Western blot analysis

The Western blot assay was used as described previously [29]. The amount of protein loaded per lane was 100 μg. Cell lysates were separated on an analytical 10% SDS-PAGE gel and transferred onto polyvinylidene difluoride (PVDF) membranes. Non-specific binding was blocked by incubation with 5% skim milk. Antibodies against human NLK, β-catenin (1:1000 dilution, Abcam), H3K27me3 (1:1000 dilution, Cell Signaling Technology), and β-actin (1: 4000 dilution, Santa Cruz) were used as the primary antibodies.

Rabbit or mouse IgG antibodies coupled to horseradish peroxidase (Santa Cruz) were used as the secondary antibodies. Antibody-labeled protein bands on the membranes were detected with a G: BOX iChemi XT chemiluminescence and fluorescence imaging system (Syngene). Band densities were determined using ImageJ software.

### RNA immunoprecipitation (RIP)

RIP assays were performed according to the manufacturer’s instructions (RIP kit, BersinBio). Five million cells were washed once in cold PBS and then pelleted. The pellet was then resuspended in 1.5 ml of polysome lysis buffer (includes DTT, an RNA inhibitor, and protease inhibitor cocktail) and incubated on ice with frequent vortexing for 10 min. The lysate was obtained by centrifugation at 16000 g for 10 min. A primary antibody or normal rabbit IgG was added, and the samples were incubated for 4 h at 4 °C. The samples were washed three times with polysome lysis buffer and twice in wash buffer. The beads were resuspended in polysome elution buffer and treated with proteinase K at 50 °C for 30 min. RNA samples were extracted with an equal volume of a phenol: chloroform: isoamyl alcohol mixture and used for qPCR. The primer for HOTAIR detection F-ATAGGCAAATGTCAGAGGGTT, R-TCTTAAATTGGGCTGGGTC.

### Chromatin isolation by RNA purification (CHIRP)

ChIRP experiments were performed using the ChIRP Kit (BersinBi). Complementary DNA oligonucleotides against HOTAIR were constructed using BLAST and 3′-end biotinylated to capture DNA–RNA hybrids on streptavidin-coated magnetic beads. Cells were incubated in 1% glutaraldehyde and were then rinsed with phosphate-buffered saline. Probes (100 pmol) were added to 1 mg of precleared chromatin and were mixed by end-to-end rotation at 65 °C for 10 min, 25 °C for 30 min, 50 °C for 5 min, and 25 °C for 90 min. The beads were resuspended in 95 μl of RNA PK buffer at pH 7.0. Next, 5-μl proteinase K was added and incubated at 50 °C for 45 min with end-to-end shaking, and Trizol reagent was used to collect RNA. For protein, 100 μl of washed/blocked streptavidin magnetic beads were added to the samples (30 min). The beads were treated with urea CHAPS buffer for 1 h at room temperature, and proteins were analyzed by Western blotting. Sequences of biotin probes were listed in Additional file 1: Table S1.

### Chromatin immunoprecipitation (CHIP)

ChIP assays were performed according to the manufacturer’s instructions (EZ-ChIP Chromatin Immunoprecipitation Kit, Upstate, Merck Millipore Co., Billerica, MA, USA). Cells (2 × 10^5^ cells/dish) were treated with 1% formaldehyde in medium for 10 min at room temperature. Glycine (1.25 M) was added to each dish at a final concentration of 0.125 M, mixed well, and incubated for 5 min at room temperature. Cells were then washed twice in cold PBS and then pelleted. The pellet was resuspended in 1 ml of SDS lysis buffer on ice and sonicated (3 s on and 9 s off at 30% maximum power for a total of 20 min). Chromatin samples were obtained by centrifugation at 13000 g for 10 min at 4 °C. The indicated primary antibody or normal rabbit IgG was added, and the samples were incubated overnight at 4 °C with rotation. The samples were then incubated with protein G agarose for 1 h at 4 °C. The Protein G Agarose-antibody/chromatin complex was washed with the following buffers (in order): low-salt immune complex wash buffer, high-salt immune complex wash buffer, LiCl immune complex wash buffer, and TE buffer. The protein/DNA complexes were eluted with elution buffer (1% SDS and 0.1 M NaHCO_3_) and incubated at room temperature for 15 min. Reversal of protein/DNA complex crosslinks was performed by adding 5 M NaCl and incubating at 65 °C for 4–5 h, followed by treatment with RNase A and Proteinase K to cleave the RNA and proteins. DNA was purified with spin columns, and the purified DNA samples were then analyzed by PCR with specific primers. GAPDH served as the control.

### Electrophoretic mobility shift assay (EMSA)

Cell nuclear extracts were applied to RNA-binding assays using the Nuclear and Cytoplasmic Extraction Kit according to the manufacturer’s protocol. Biotin-labeled wild-type HOTAIR oligonucleotides (5′-GUGGGGAGUGGAGAGAGGGAGCCCA-3′) or mutated HOTAIR oligonucleotides (HOTAIR-36U46A: 5′-GUGGGUAGUGGAGAGAGGGAGCCCA-3′; HOTAIR 36G46G: 5′-GUGGGGAGUGGAGAGGGGGAGCCCA-3′; HOTAIR 36U46G: 5′-GUGGGUAGUGGAGAGGGGGAGCCCA-3′) were used for the EMSA. The reaction mixture was loaded on a 5% native polyacrylamide gel in 0.5% Tris-borate-EDTA and blotted onto membranes (Millipore). After 7 min of UV cross-linking, the interaction between the biotin-labeled HOTAIR oligonucleotides and the EZH2 protein was detected using a chemiluminescent EMSA kit (Beyotime) with a streptavidin-HRP conjugate and a chemiluminescent HRP substrate (Millipore).

### Wound healing assay

Approximately 2 × 10^5^ cells per well were plated onto 6-well plates. A linear scratch/wound was made on the cell monolayer using a sterile pipette. Photomicrographs of live cells were captured at × 100 magnification, and the distance migrated was determined within an appropriate time.

### Matrigel invasion assay

The Matrigel invasion assay was performed in 24-well transwell culture plates. Briefly, 40 μl of Matrigel (1 mg/ml, BD) was applied to 8-μm polycarbonate membrane filters. Approximately 5 × 10^4^ cells were resuspended and then seeded onto the upper chambers of 24-well transwell plates containing FBS-free medium for 24 h at 37 °C. The medium in the lower chamber was replaced with complete growth medium containing 10% FBS, and the cells were cultured for 48 h at 37 °C. Non-invading cells were removed from the upper surface of the invasion membrane, and those on the lower surface were stained with crystal violet. The average number of cells per field was determined by counting the number of cells in six random fields per well. Cells were counted in four separate fields in three independent experiments.

### Orthotopic nude mouse models and treatment

BALB/c nude mice (4–6 weeks old) were purchased from the Animal Center at the Cancer Institute of the Chinese Academy of Medical Science (Beijing, China). All experimental protocols were approved by the Tianjin Medical University Animal Care and Use Committee. To establish orthotopic models, MDA-MB-231 cells transduced with luciferase lentivirus were injected into the mammary fat pads of each nude mouse. The mice were randomly assigned to six groups (*n* = 8 per group). Twenty-one days after implantation, PBS or ADQ (15 mg/kg) was administered by intraperitoneal injection every 2 days for 21 days. The mice were imaged for luciferase activity once per week. The mice were sacrificed on days 35 and 42, and their heart, lungs, liver, spleen, and kidneys were removed for bioluminescence imaging.

### Hematoxylin and eosin staining

Paraffin-embedded tissue sections were subjected to H&E staining. After blocking with xylene, sections were deparaffinized and rehydrated with ethanol and excess deionized H_2_O. Stain hematoxylin for 3 min followed by eosin staining and dehydration. Coverslip slides using neutral gum. Sections were visualized under a light microscope.

### Analysis of metastasis

Six weeks after injection, all mice were killed and the number of surface metastases per lung was determined under a dissecting microscope. The left lower lobe of the lung was isolated, fixed, paraffin-embedded, and coronally sliced into 4-μm thicknesses. The tissue sections were stained with H&E. An anatomical microscopic metastasis quantitation was performed by counting the metastasis nodes with different diameters on the surface of all lobes of lungs for each mouse by two observers in a blinded fashion.

### Statistical analyses

SPSS 16.0 (IBM, USA) was used for all calculations. All values are presented as the mean ± SD. Statistical comparisons between two groups were performed using Student’s *t* test. Differences among groups were determined by two-way ANOVA followed by Dunnett’s test. Significance was set to *P* < 0.05, *P < 0.05, and **P < 0.01.

## Additional file

Additional file 1: **Table S1.** Sequence of biotin probes of HOTAIR for CHIRP assay. **Table S2.** Evaluation of potent small molecule inhibitors of HOTAIR. The chemical structure and docking results of each compound were measured. **Table S3.** Structure-activity relationship studies. The chemical structure of the structural analogs of ADQ was shown. **Figure S1.** Nuclear magnetic resonance image of ADQ. **Figure S2.** ADQ specifically blocks the HOTAIR/EZH2 interaction. RIP assays were performed to detect the binding efficiency of the MALAT1, HOTAIRM1, KCNQ1OT1, HOXA11, and XIST fragment in combination with EZH2 proteins. Levels of retrieved lincRNA in immunoprecipitates were determined by qPCR in U87 cells. IgG was the negative control. Data were represented as mean ± s.d.; *n* = 3 independent experiments. **P < 0.001, *P < 0.05. Two-tailed unpaired Student’s *t* test. **Figure S3.** Knock down HOTAIR inhibited cell growth in vivo. Representative pseudocolor bioluminescence images of mice treated with shHOTAIR, indicating that ADQ treatment resulted in cell growth inhibition similar to that in the HOTAIR knockdown group. **Figure S4.** ADQ enhanced the mRNA expression and protein levels of ZHX2, another target of HOTAIR. (a) MRNA levels of ZHX2 were measured in U87 cell lines via qRT-PCR after treatment with ADQ. (b) Protein levels were detected by Western blotting. (c) Representative images of the immunohistochemical staining of ZHX2. (DOC 2147 kb)

## Abbreviations

CHIRP: Chromatin isolation by RNA purification
EMSA: Electrophoretic mobility shift assay
HOTAIR: HOX antisense intergenic RNA
NLK: Nemo-like kinase
PRC2: Polycomb repressive complex 2
RIP: RNA immunoprecipitation
