# Supplementary material for: Targeted design and identification of AC1NOD4Q to block activity of HOTAIR by abrogating the scaffold interaction with EZH2
Source: Clin Epigenetics. 2019 Feb 14;11:29. doi: 10.1186/s13148-019-0624-2 (PMC6376746; doi:10.1186/s13148-019-0624-2)
Supplement: Supplementary file 1 — Table S1. Sequence of biotin probes of HOTAIR for CHIRP assay. Table S2. Evaluation of potent small molecule inhibitors of HOTAIR. The chemical structure and docking results of each compound were measured. Table S3. Structure-activity relationship studies. The chemical structure of the structural analogs of ADQ was shown. Figure S1. Nuclear magnetic resonance image of ADQ. Figure S2. ADQ specifically blocks the HOTAIR/EZH2 interaction. RIP assays were performed to detect the binding efficiency of the MALAT1, HOTAIRM1, KCNQ1OT1, HOXA11, and XIST fragment in combination with EZH2 proteins. Levels of retrieved lincRNA in immunoprecipitates were determined by qPCR in U87 cells. IgG was the negative control. Data were represented as mean ± s.d.; n = 3 independent experiments. **P < 0.001, *P < 0.05. Two-tailed unpaired Student’s t test. Figure S3. Knock down HOTAIR inhibited cell growth in vivo. Representative pseudocolor bioluminescence images of mice treated with shHOTAIR, indicating that ADQ treatment resulted in cell growth inhibition similar to that in the HOTAIR knockdown group. Figure S4. ADQ enhanced the mRNA expression and protein levels of ZHX2, another target of HOTAIR. (a) MRNA levels of ZHX2 were measured in U87 cell lines via qRT-PCR after treatment with ADQ. (b) Protein levels were detected by Western blotting. (c) Representative images of the immunohistochemical staining of ZHX2. (DOC 2147 kb) [file 13148_2019_624_MOESM1_ESM.doc]

**Table.S1** Sequence of biotin probes of HOTAIR.

| Number | Sequence |
| --- | --- |
| 1 | tgtggaagctttcggatcaa |
| 2 | ttagggacctgagggtctaa |
| 3 | aaatccgttccattccactg |
| 4 | aataaagacgcccctccttc |
| 5 | tttcagccttttctctgcca |
| 6 | ggtgtaattgctggtttagg |
| 7 | taaacctctgtctgtgagtg |
| 8 | aggtttttccagcgttctct |
| 9 | attaattagcgcctcccagt |
| 10 | ctgtttgggcctcctaaaat |
| 11 | tgttcctctcaaattccgga |
| 12 | tgtgctgccagttagaaaag |
| 13 | ctgtgtctacatgcatcact |
| 14 | catacctacccaatgtatgg |

**Table.S2** Evaluation of potent small molecule inhibitors of HOTAIR. The chemical structure and docking results of each compound were measured.


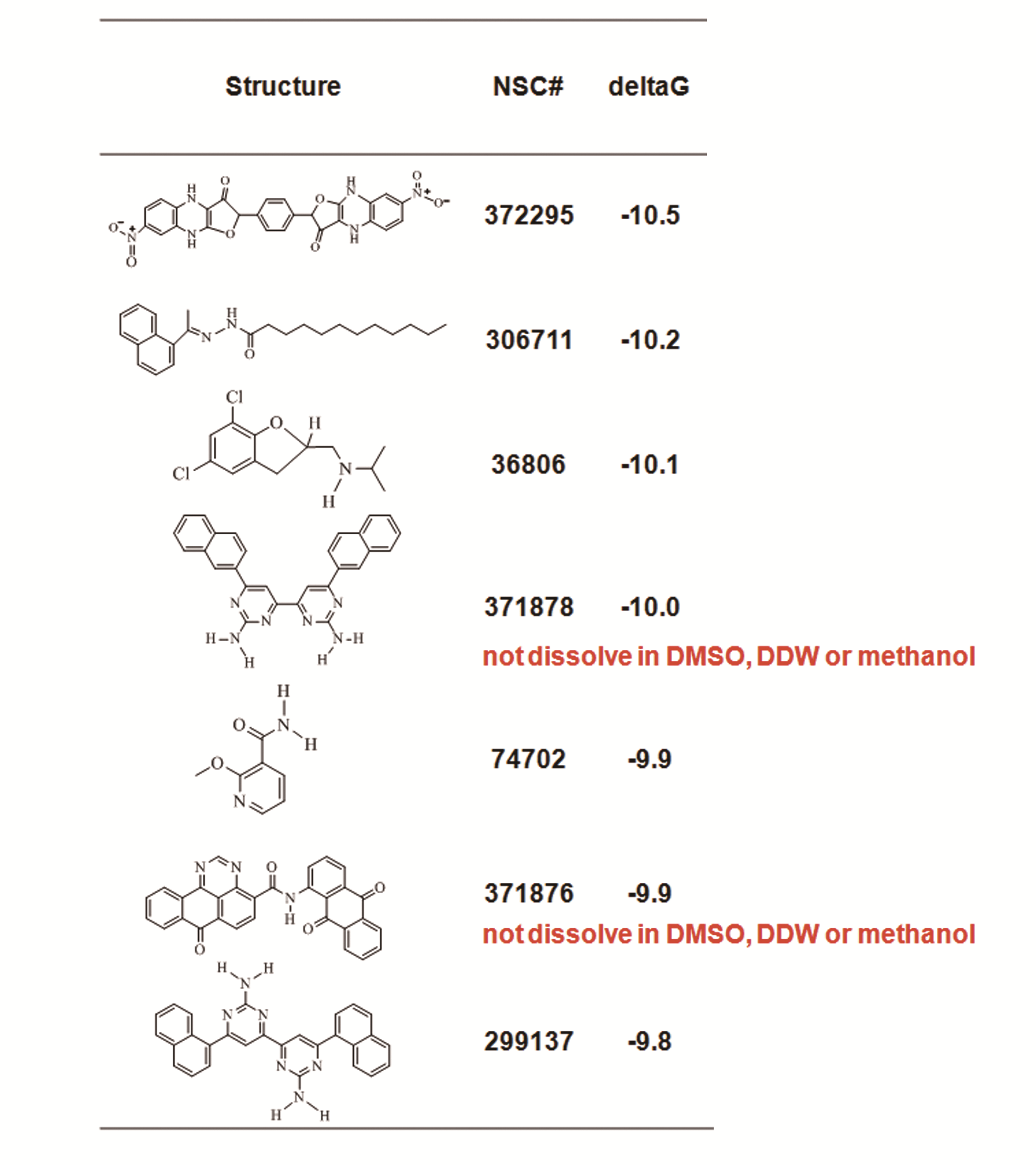


**Table.S3** Structure Activity Relationship Studies. The chemical structure of the structural analogs of ADQ was shown above.


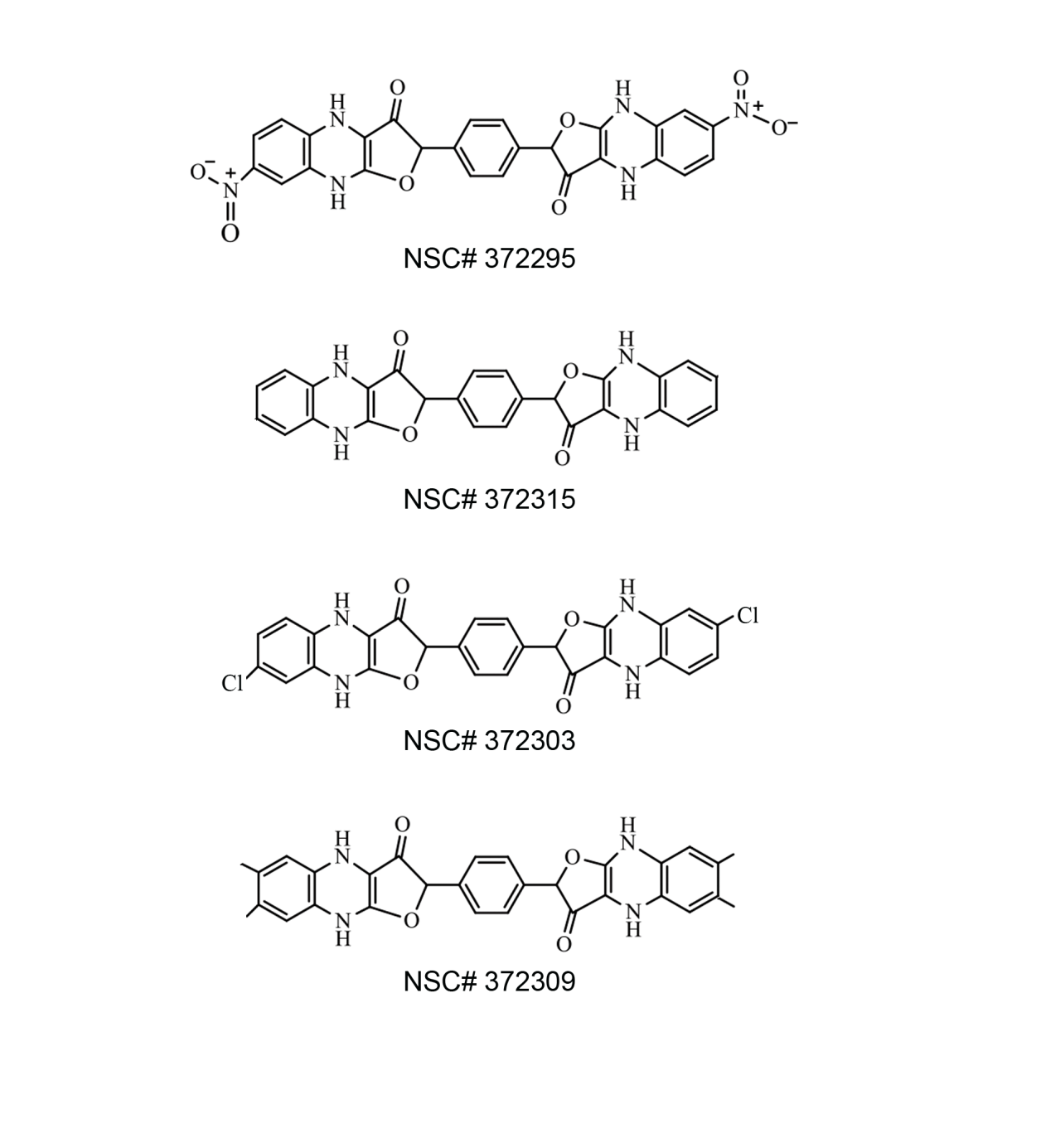


*^1^H Spectrum*


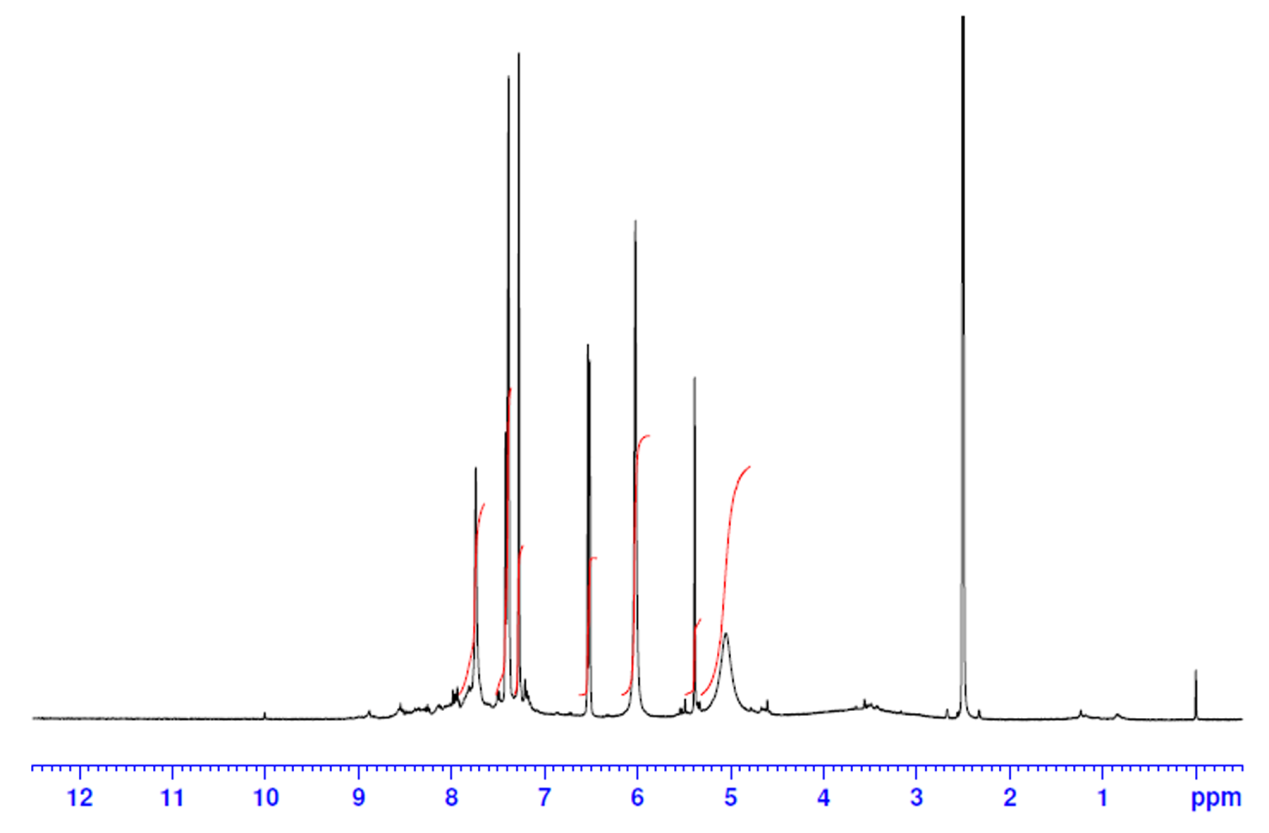


*^13^C Spectrum*


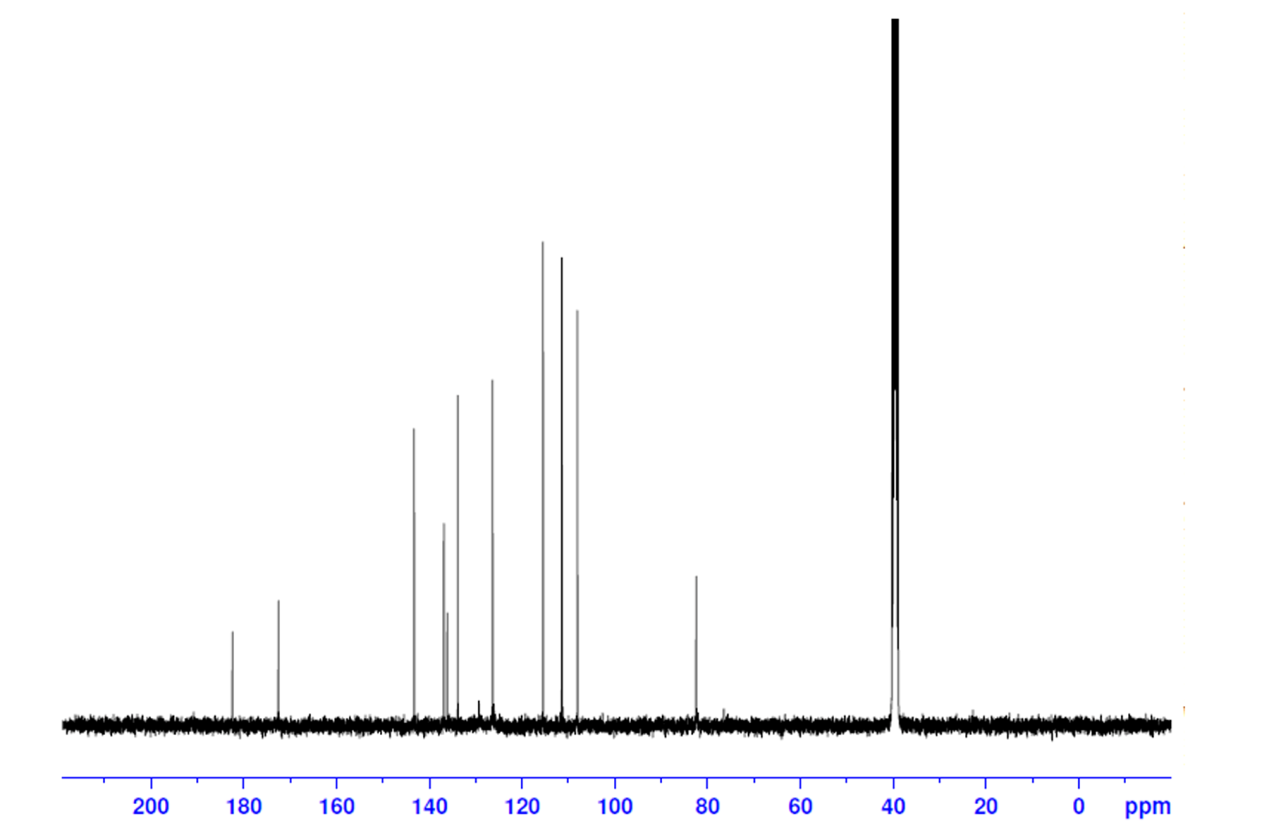


**Fig.S1** Nuclear Magnetic Resonance image of AC1NOD4Q.


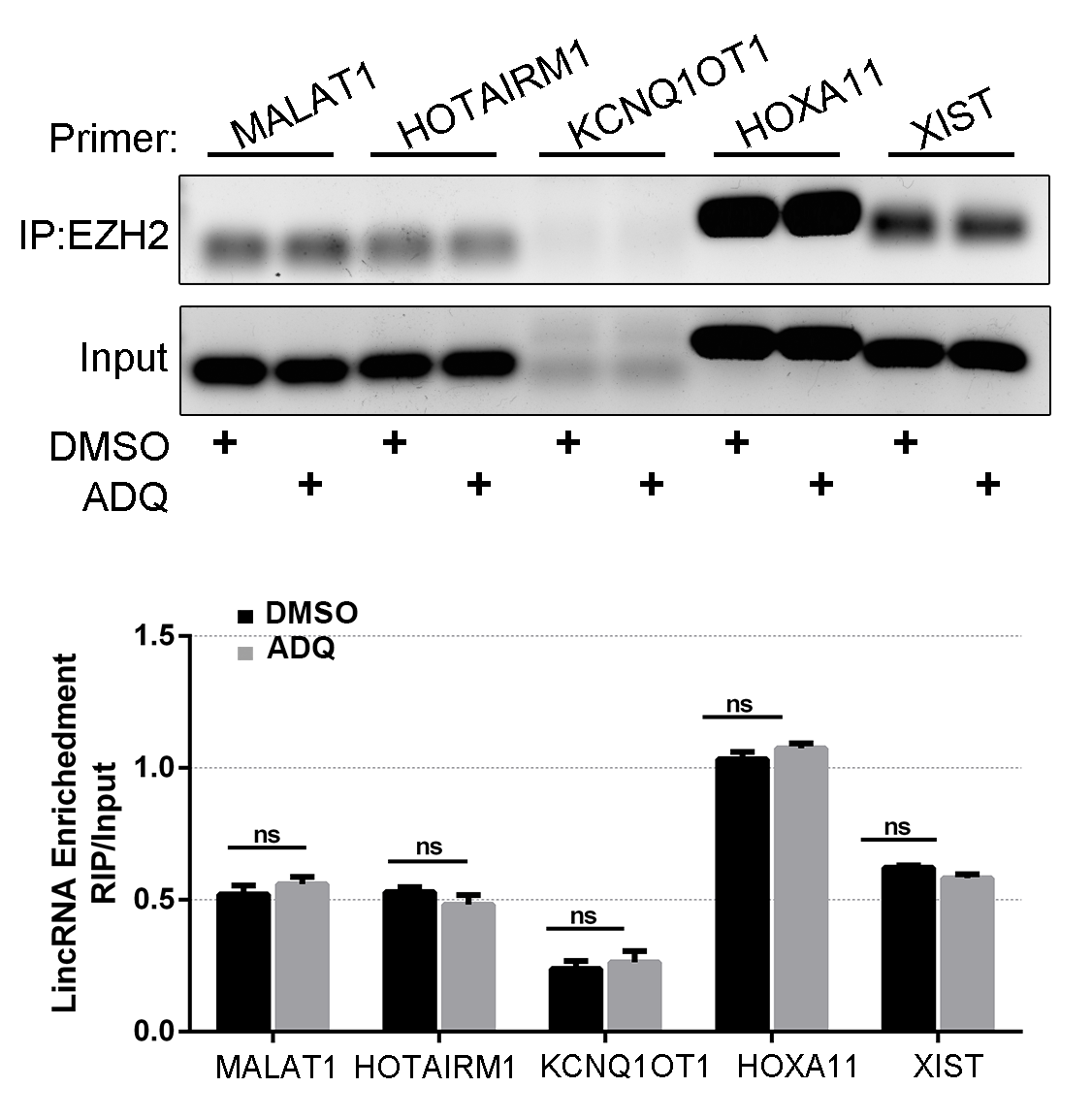


**Fig.S2** ADQ specifically blocks the HOTAIR/EZH2 interaction. RIP assays were performed to detect the binding efficiency of the MALAT1, HOTAIRM1, KCNQ1OT1, HOXA11 and XIST fragment in combination with EZH2 proteins. Levels of retrieved lincRNA in immunoprecipitates were determined by qPCR in U87 cells. IgG was the negative control. Data were represented as mean ± s.d.; n = 3 independent experiments. **P<0.001, *P<0.05. two-tailed unpaired Student’s t-test.

**
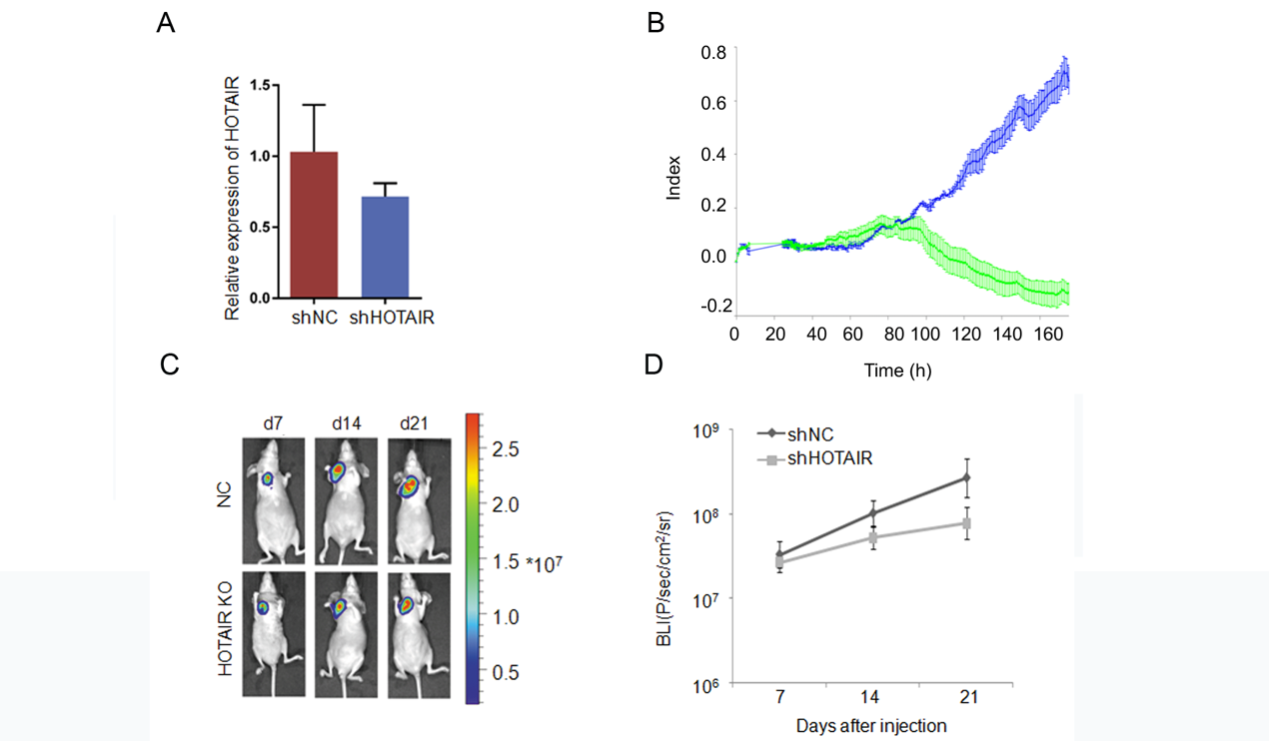
**

**Fig.S3** Knock down HOTAIR inhibited cell growth in vivo. Representative pseudocolor bioluminescence images of mice treated with shHOTAIR, indicating that ADQ treatment resulted in cell growth inhibition similar to that in the HOTAIR knockdown group.


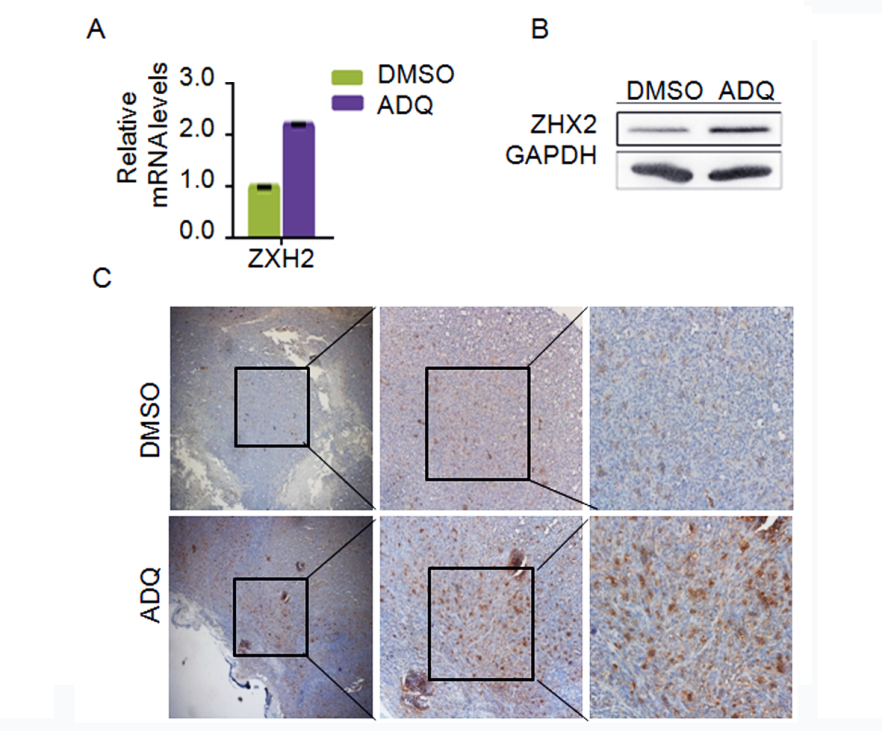


**Fig.S4** ADQ enhanced the mRNA expression and protein levels of ZHX2, another target of HOTAIR. **(a)** MRNA levels of ZHX2 were measured in U87cell lines via qRT-PCR after treatment with ADQ. **(b)** Protein levels were detected by Western blotting. **(c)** Representative images of the immunohistochemical staining of ZHX2.
